# ‘It benefits patient care’: the value of practice-based IPE in healthcare curriculums

**DOI:** 10.1186/s12909-020-02356-2

**Published:** 2020-11-12

**Authors:** Noreen O’Leary, Nancy Salmon, Amanda M. Clifford

**Affiliations:** School of Allied Health, Faculty of Education & Health Sciences, Health Research Institute, University of Limerick, Limerick, Ireland

**Keywords:** Interprofessional education, Activity theory, Hofstede’s cultural dimensions, Qualitative case study

## Abstract

**Background:**

Practice-based interprofessional education (IPE) is essential to prepare students for collaborative working. Pockets of practice-based IPE are integrated into healthcare curriculums in some regions. Yet practice-based IPE is not globally valued as a key element of healthcare curriculums. As students and clinical educators are key stakeholders, this study presents a case example of their experiences in a country where practice-based IPE is at an emergent stage. Their experiential knowledge generated important insights into how practice-based IPE is perceived. This learning can be applied, both locally and further afield, by those seeking to embed practice-based IPE in their placement curriculums.

**Methods:**

A qualitative case study was conducted at a school of allied health and partner placement sites in Ireland. Data collection comprised two participant observations, 13 interviews and 12 document analyses. Inductive thematic analysis and deductive framework analysis, underpinned by activity theory and Hofstede’s cultural dimensions, informed data analysis and interpretations.

**Results:**

Participants are grappling to establish the value of practice-based IPE, illustrated in three themes: clarifying the concept of practice-based IPE, mapping IPE activities and diversifying interprofessionalism. First, ambiguous conceptualisation of why and how to implement practice-based IPE was identified. Highlighting how practice-based IPE improved patient care and safety created a clear rationale for implementation. It was also helpful to demonstrate how adaptations to existing practice education models, rather than entirely new models, could achieve high-quality practice-based IPE. Second, the positioning of practice-base IPE in the placement curriculum was unclear. Overt mapping of practice-based IPE activities onto learning outcomes within assessment tools enhanced its value within practice education. Third, varying levels of professional engagement were noted, perpetuating stereotypes. Creating diverse educator networks and embedding practice-based IPE in organisational strategy may incentivise engagement across a greater range of professions.

**Conclusions:**

Implementing these recommendations could enhance the value of practice-based IPE and optimise student preparation for collaborative working. Practice-based IPE remains a complex model and the trajectory of embedding in healthcare curriculums will differ globally.

**Supplementary Information:**

The online version contains supplementary material available at 10.1186/s12909-020-02356-2.

## Background

Interprofessional collaboration (IPC) is necessary for optimal patient care and outcomes [1]. Therefore, students require appropriate preparation to enter the workforce as collaborative-ready, patient centred practitioners. There are many ways of preparing students for IPC, subsumed by the umbrella term interprofessional education (IPE). IPE can be broadly categorised as classroom-based, simulated and practice-based. Practice-based IPE requires students from two or more professions working and learning together at the same placement site [2]. Location at clinical sites provides unique learning opportunities [3] as students apply theory to practice [4], experience IPC first-hand [5] and commence socialisation into clinical teams [6, 7]. Indeed, healthcare professionals whose training included IPE cite practice-based IPE as the most meaningful IPE input in terms of clinical practice [8, 9]. However, understanding of student and clinical educator experiences as practice-based IPE becomes embedded in a curriculum is relatively limited. Therefore, it is critical to explore this process in depth, to advance integration of practice-based IPE and optimise student preparation for IPC.

There are challenges specific to integrating practice-base IPE that differ from those relating to classroom IPE. Beyond the well documented logistical complexities [10], practice-based IPE involves tackling sensitive issues such as professional stereotypes and role boundaries in often demanding clinical settings [11] where patient safety and wellbeing are the primary focus [7]. Educators at clinical sites are primarily practicing clinicians [12] and can sometimes lack educator specific training even uniprofesionally [13, 14]. IPE facilitation is perceived as a complex role for educators [15] and targeted training is rare [3, 16]. Consequently, clinical educators may be reluctant to become involved in practice-based IPE. Additionally, all practice education must ensure students achieve competencies required by their professional regulatory body [17]. As such, practice-based IPE is a complex practice education model.

Furthermore, practice-based IPE occurs at the interface of education and frontline health services, both of which are influenced by the social and cultural context [18]. Therefore, experiences of embedding practice-based IPE likely differ internationally. For instance, interprofessional training wards at acute hospitals are well established in Scandinavian countries [19], while rural and remote healthcare activities are often reported in Australia [20]. Geographical [21] and specific healthcare needs and resources [22] likely influenced the approach taken in these regions. Globally, long-term funding for practice-based IPE is an on-going challenge [23] and many practice-based IPE projects do not extend beyond pilot or short-term initiatives [24]. This has stimulated growing interest in relatively low resource activities such as case-based tutorials [25–27]. Currently, practice-based IPE is not cohesively integrated into healthcare curriculums globally [28].

Theory provides a crucial anchor when seeking a nuanced understanding of how students and clinical educators experience this complex model [29]. Activity theory is suitable for unpicking the interacting factors influencing practice-based IPE, as it focuses on how people engage within rule-governed systems and use tools to achieve objectives in real-life circumstances [30]. During practice-based IPE, distinct students and clinical educator activity systems temporarily coalesce [31]. Within and across these activity systems tensions can arise, for example between differing objectives [32] (further detail can be found in Additional File 1). Given the seismic changes occurring in health and education spheres globally due to the COVID-19 pandemic [33], it is perhaps more crucial than ever to analyse how national socio-political contexts intersect with implementing changes to healthcare education models such as practice education [34]. Hofstede’s cultural dimensions theory [35] offers one interpretation for how national culture can influence values and behaviours [34]. Hofstede posits that as people are exposed to national cultures from birth, these traits are more ingrained than workplace culture, which is more transient and acquired later in development [36]. Cultural trends considered by Hofstede include attitudes to democracy, individualism or collectivism, tradition and achievement as well as long and short term planning and enjoyment of life [35] (Further information can be found in Additional File 2). Regarding practice-based IPE this theory can contribute to understanding how and why IPE has evolved differently across countries.

The aim of this research was to develop an in-depth treatise of student and clinical educator experiences while seeking to embed practice-based IPE in the curriculum. To this end the following objectives were developed:

- To document the practice-based IPE experiences of students and clinical educators affiliated with one university.
- To explore the context in which these activities developed.
- To develop recommendations supporting sustainability and growth of practice-based IPE activities with applicability beyond the research site.

As such this paper will contribute to the discussion on how to embed practice-based IPE as a valued aspect of health professions education, providing signposts for stakeholders including clinical educators and accrediting bodies.

## Methods

This qualitative case study facilitated in-depth exploration of practice-based IPE within the parameters of a specific case [37], consisting of practicum sites connected to an Irish university. Five allied health professional qualification programmes are offered by the university. Students attend diverse placements including hospital, community care and rehabilitation sites. This research forms one phase of a larger doctoral study at the same site. A previous study has explored the experiences of university-based educators involved in developing and coordinating practice-based IPE (under review). Ethical approval was provided by the university and placement site Research Ethics Committees. The Standards for Reporting Qualitative Research were used to report key features of the research process [38] (Additional File 3).

### Data collection

Data collection occurred from November 2019 to April 2020. However, the foundations for this phase, including familiarity with placement structures and access to potential gatekeepers, were in place from previous research at the site, which began in 2017. Methodological triangulation was used to enhance data collection validity [39] and credibility of findings [40].

#### Observations

Participant observations were conducted to allow the researcher to develop a first-hand and socially contextualised understanding of practice-based IPE [41]. Using a specifically designed template (Additional File 4), the first author observed interprofessional tutorials (*n* = 2) over 5 h. Participants included seven clinical educators and 17 students. Five professions were represented - nursing, occupational therapy, physiotherapy, radiography and speech and language therapy.

#### Interviews

Semi-structured interviews (*n* = 13) were carried out by the first author to facilitate exploration of individual experiences and perspectives [42]. Interview length ranged from 26 to 42 min, with a median length of 33 min. Participants were clinical educators (*n* = 4), current students (*n* = 7) and recent graduates (*n* = 2). Four professions were represented - occupational therapy, physiotherapy, dietetics, and speech & language therapy. Interview guides were informed by observations, literature, and theory (Additional File 4).

#### Document analysis

Relevant documents (*n* = 12) were analysed in conjunction with observations and interviews [40] to facilitate comparison of stated policy and guidelines with participant experiences [43] and to generate further lines of inquiry. Documents included profession-specific competency forms and interprofessional education resources.

### Data analysis

**Fig. 1:**
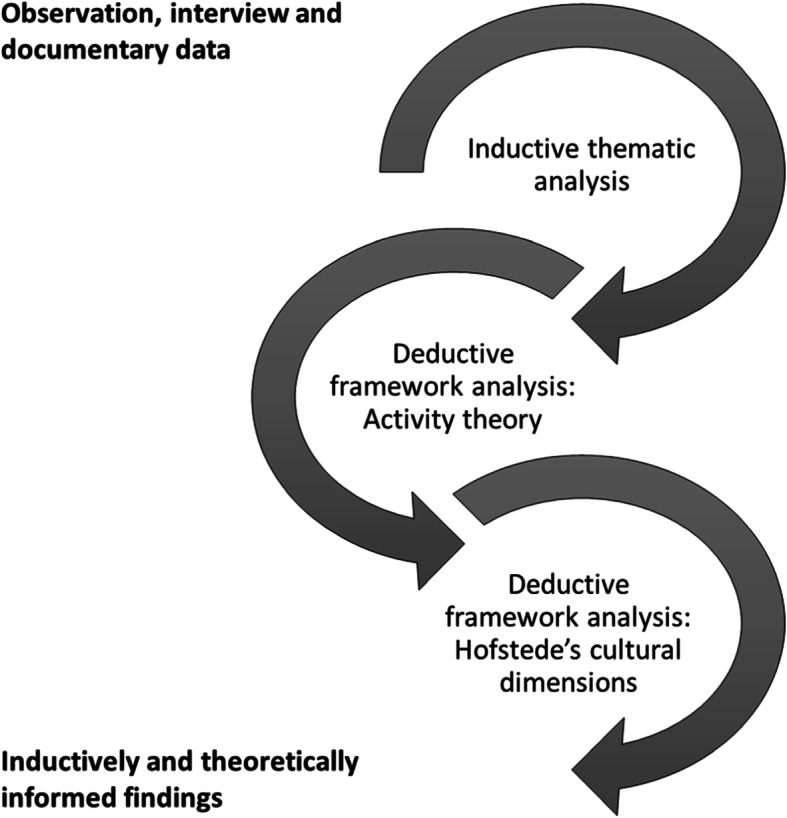
Data analysis process

Observation, interview and documentary data were imported into NVivo12 software to support data management [44]. Thematic and framework analyses were used to interpret data as per Fig. 1. Analytical pluralism was adopted to achieve more nuanced data interpretations than would be achieved through use of either approach singularly [45] and to limit interpretive bias [46]. Thematic analysis was used to inductively code and interpret participant data and develop initial themes [47]. A deductive framework analysis was then used to analyse participant data using a priori codes [48] from activity theory [49] and Hofstede’s cultural dimensions [35]. Initially, the first and second author individually coded a subset of three transcripts. This enhanced the comprehensiveness of the initial inductive coding framework and refined the application of the theory-based deductive framework. Sample data analysis can be found in Additional File 5. The approaches chosen were philosophically compatible [50], as neither is aligned to a specific epistemological perspective and both focus on generating themes [48, 51]. Reflexive memoing [52] along with ongoing author and advisory panel discussions enabled exploration and resolution of divergent interpretations [48].

**Fig. 2:**
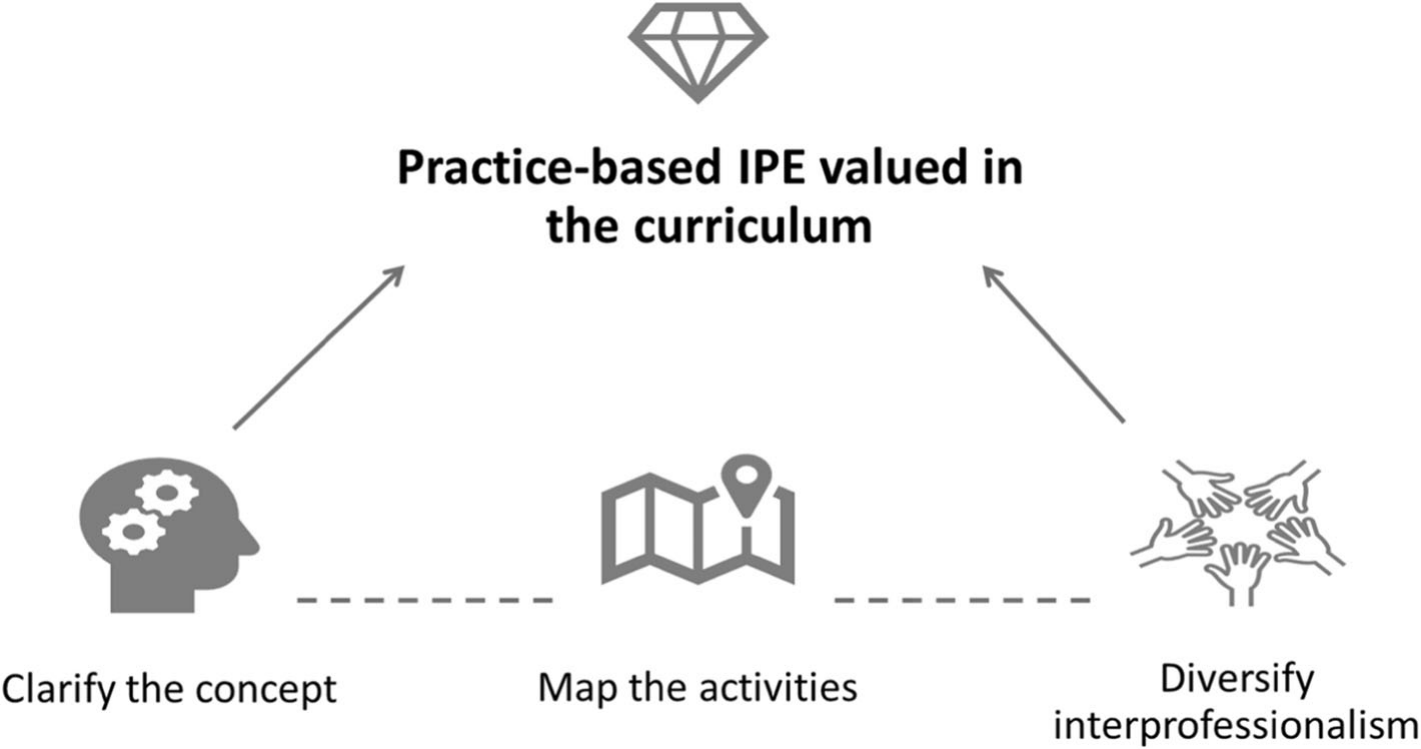
Developing the value of practice-based IPE

## Results

We begin this section by framing the context in which participants reported they experienced practice-based IPE. Students from each programme typically complete four blocks of placement. Between placements, students complete five interprofessional academic modules, designed to establish foundations for collaborative working. For example, shared attendance at sessions on topics such as professional documentation, infection prevention and control and ethics. Students subsequently engage in interactive interprofessional modules where they develop interprofessional management plans for hypothetical complex cases.

Operationally, placement timetables were aligned to maximise opportunities for practice-based IPE. Students engage in practice-based IPE at any stage of their programme. The experiences included in this study captured all placement stages, from initial to final placements. The content and format of practice-based IPE is decided locally by clinical educators, guided by clinical needs and facilitation resources available at the placement site. Placement handbooks outline opportunities for practice-based IPE and signpost clinical educators to useful resources, such as a practice-based IPE resource pack developed by the university practice education team. Those involved in practice-based IPE depend on the professions available at the site at the time and agreement by educators from programmes to enable students to become involved.

Participant data reflected a situation of fledgling practice-based IPE at an early stage of integration into the practice education curriculum. Participants are grappling with cultivating the value of practice-based IPE, represented in three key themes (Fig. 2):

- Clarifying the concept of practice-based IPE
- Mapping practice-based IPE activity
- Diversifying interprofessionalism

In activity theory terms, these themes reflect sources of tension within the systems of practice education as participants sought to embed practice-based IPE.

**Conceptualising practice-based IPE.**

At a conceptual level, participants reported equivocation regarding two key issues, the rationale for practice-based IPE and the process of establishing it.

Both students and educators experienced uncertainty about why practice-based IPE was needed:

*I was baffled as to why it’s required, or who these people are.* [Student 7]

*Some of the nurses didn't even know what IPE was.* [Clinical Educator 1]

For example, educators reported that many colleagues perceived practice-based IPE as a purely educational activity and did not link it to improved clinical practice and patient outcomes:

*The one thing that made [the nurses] open their eyes a little bit was when we said, "No actually there's evidence, they say it benefits patient care and patient outcomes"… it wasn't, ‘all students think it's great’ … this is what the benefit is.* [Clinical Educator 1]

In a similar vein, when students experienced practice-based IPE it deepened their understanding of its contribution to patient care:

*I think now I have an understanding of how important interprofessional working is, I could advocate for that a bit more, having seen it*. [Student 3]

Activity theory highlights that activity is objective driven. Activity that aligns with core objectives of healthcare is likely to be perceived as valid. As in this example, spotlighting the impact of practice-based IPE on improved patient safety and care added validity.

Students and educators expressed concern that practice-based IPE was resource intensive, creating additional work for clinical educators and reducing student time for uniprofessional activity.

*I think it would be a mistake to make it [IPE] a big job because I think it would turn people off and it feels forced then, when it should just be kind of a case discussion*. [Clinical Educator 1]

Indeed, feedback from graduates and educators who experienced practice-based IPE illustrated that small-scale activities, building on existing clinical activity provided impactful learning opportunities. As a case example, during an acute hospital placement two graduates each worked with a student from another profession, to jointly assess a patient, develop an interprofessional management plan and present their findings to their clinical educators:

*What we did for our project, it wasn't overly complicated. It had nice structure to it, but it wasn't complicated.* [Graduate 1]

The structure came from a template contained in the IPE resource pack provided by the university. Key features of this template were sharing information about each profession, negotiating, and reflecting on learning about working with other professions. Graduates felt learning would not have been as impactful without this tool:

*If it was just passively going in, observing each other without really thinking about what we were trying to get out of it.* [Graduate 2]

From the activity theory perspective, the template provided a tool for students to divide labour in pursuit of the shared objective of patient care while also prompting critical reflection. Small-scale activities such as this can lead to meaningful outcomes, in terms of student’s interprofessional interactions. For example, clinical educators and students experienced increased interprofessional communication following a two-hour interprofessional tutorial:

*I actually had a number of students approaching me … and say, "Can I ask you a question about this patient?... I don't think she'd have approached me without having done those sessions. I haven't come across that before.* [Clinical Educator 2]

Students themselves reflected that it was working together during tutorials which facilitated future communication and interactions

*I was less cautious about approaching the other professions, so I really noticed that actually after the IPE tutorial … it kind of broke down the barrier.* [Student 5]

### Mapping interprofessional activity

In this theme we explore how practice-based IPE is currently mapped onto placement curriculums. Overall students and educators asserted that while IPE was important, their priorities, and thus activity, during practice education is guided by the competency forms on which students are graded.

*On placement, you're being marked, you're being graded and it's worth a lot to your degree.* [Student 2]

There was a prevailing sense of ambiguity about the place of practice-based IPE in the placement curriculum and assessment:

*I think it is kind of an unwritten rule that on your placements you will do sessions with other professionals* [Student 8]

Each profession is assessed using a different competency tool. Most competencies refer to uniprofessional activities with some lending themselves to practice-based IPE. However, the wording of the latter competencies allows considerable interpretative latitude, for example:

*Contributes effectively as a team member; build collaborative working relationships* [53]

This was confirmed by student recollections of variable practice-based IPE experiences regarding type and level of interprofessional collaboration:

*My first placement wasn't a multidisciplinary setting … [so] you were graded on your communication with everyone else … even with the receptionist and everybody else in general, they looked at that as a whole*. [Student 4]

The most common practice-based IPE opportunities were acute placement sites, supported by co-location of professions and patient needs. This took the form of interprofessional tutorials, case presentations and joint assessments. Many students identified missed opportunities for practice-based IPE:

*On my last placement, there were other students there … I think there was one day a week we were in the same building … even if there was a half an hour a week just set aside for group talk or something like that... talk over or plan something.* [Student 8]

Across the board there were variable interpretations as to how interprofessional activity informed student assessment. For example, the following two students reported contrasting experiences of the same practice-based IPE activity and its link to their assessment:

*The practice educator said before the sessions you're not being assessed on this.* [Student 5]

*The educator was observing [the tutorial] and she even drew back to that when we were completing the form then that she'd seen me recognize the role of the other professionals.* [Student 3]

To begin addressing these inconsistencies clinical educators reflected that making explicit links between practice-based IPE and professional competency assessment strengthened alignment between the activity and assessment and created a clear rationale for the activity, thus enhancing its value:

*We're very clear and we can tell them beforehand, these are the competencies, that it's going to help you to progress in … there's a good reason why we're asking you to do this.* [Clinical Educator 3]

Participants acknowledged a lack of guidance from higher level bodies such as the professional regulator regarding practice-based IPE contributed to ambiguity:

*CORU* [professional regulator] *set clinical expectations for students. So maybe that's something to think about… clinical competencies that specifically relate to working as part of a team or something that you could demonstrate that in [interprofessional] sessions*. [Student 5]

Indeed, from the lens of activity theory, articulating practice-based IPE expectations more explicitly within the regulatory and competency tools mediating placement activity could support integration of practice-based IPE. Moreover, clearly mapping interprofessional activities onto competencies increases clarity about the function of the activity and the intended results. Maximising clarity is a useful approach when introducing any new practice. This strategy is especially beneficial in countries where uncertainty avoidance is culturally important. This in turn may increase the perceived value of practice-based IPE.

### Diversifying interprofessionalism

We found that involving the full range of professions in practice-based IPE is challenging. Both students and educators noted variable professional involvement:

*[It’s] dependent on people doing it out of the goodness of their hearts and their interest* [Clinical Educator 4]

*We don't have any collaboration with medics.* [Student 5]

Thus, involvement across professions relies on individual educators rather than being an integrated expectation across practice education:

*A medic involved in the medical school here he was really keen on it but then he left*. [Clinical Educator 3]

The absence of certain professions may leave professional stereotypes unchallenged. For example, medical students or educators were not involved in interprofessional tutorials observed for this research. During a group activity to develop a patient care plan one participant commented:

*Then the medic comes in and says discharge.* [Interprofessional tutorial observation 1]

The implied meaning was medics override other professions and the group response of laughter, and head nodding indicated agreement with this perspective. In their absence, the ‘us/them’ stereotype regarding one profession was perpetuated between other professions. Furthermore, student reflections highlighted that it was collective participation in practice-based IPE activities that established communication bridges with students from other professions:

*I never asked a question to one of the medical [students]. I don't know if them being at the interprofessional sessions would have made them seem like real life people ...they were in the same room at lunch, they're in the same building, but I never talked to them.* [Student 6]

Without a guided opportunity to initially engage with other professions, shared presence in clinical and social spaces did not translate to interprofessional communication and working.

Educators noted there can be a hesitancy to become involved if IPE is perceived to be the property of specific professions or people:

*If it's all coming from me then people are always going to be a bit suspicious … Why are they doing this now and what's the agenda here?* [Clinical Educator 2]

In terms of activity theory, there appeared to be poorly developed communities to support practice-based IPE. While practice education staff at the university are a clearly defined unit, this differs at clinical sites. Educators work within their own professions, links with educators in other professions are developed ad hoc by individuals:

*I met with X and she was very keen, like myself, so we decided we'd do it [IPE] and we did*. [Clinical Educator 3]

Participants felt that innovations such as practice-based IPE would be perceived as having greater value if initiated and supported by management within the healthcare organisation:

*We’re just two tutors. Whereas, if someone said, "Oh actually, we're the new managers in student education in the hospital" … then everyone is like, “this is someone who maybe can get us things or get stuff done for us" … I think if you're sending an email from a person like that, at least there's a bit of buy in.* [Clinical Educator 1]

Viewing this through Hofstede’s cultural dimensions of individualism and achievement orientation, if educators can see the benefit of involvement to their profession within their organisation they may be more positively predisposed to involvement. As such integrating practice-based IPE as an organisational priority may be advantageous in promoting practice-based IPE as valued activity across professions.

**Fig. 3:**
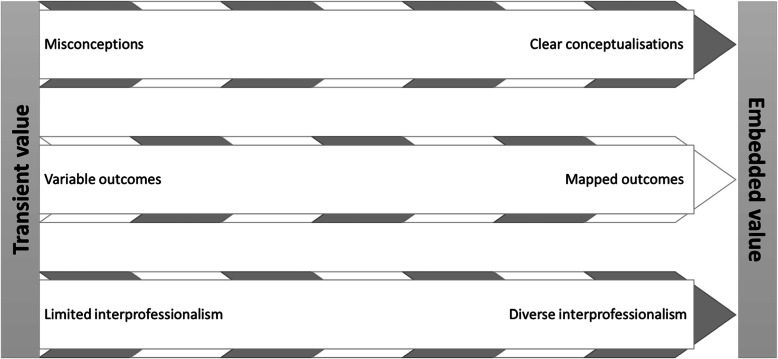
Factors influencing value of practice-based IPE

Based on the findings reported above, Fig. 3 provides an overview of how practice-based IPE can attain greater value at clinical sites and thus become more embedded in practice.

## Discussion

Practice-based IPE offers a powerful opportunity to prepare students for future collaborative practice [8]. Nevertheless, development of practice-based IPE lags behind classroom and simulation IPE [54] and requires a firmer footing in healthcare curriculums [55]. This study identified conceptualisation of practice-based IPE, mapping of IPE activities and interprofessional diversity as key features of embedding practice-based IPE. Drawing on these findings we make recommendations to enhance the value of practice-based IPE in clinical settings. The goal is not to develop a universal practice-based IPE model. This is neither practicable nor desirable given the inherent variability across placement sites [56]. Rather, learning from experiences thus far can inform future practice-based IPE initiatives and clarify the hallmarks of embedded practice-based IPE in healthcare curriculums.

In this study practice-based IPE primarily occurred at acute sites, mirroring international trends [28]. Physical co-location of students at these sites, in conjunction with the diverse clinical needs among patients likely support practice-based IPE. However, development of practice-based IPE at the level of existing primary healthcare sites could materially extend capacity and scope of practice-based IPE [57]. Moreover, considering international aims to optimise community-based care [58], it is timely to develop opportunities for community practice-based IPE. While co-location with other students can be challenging at community sites, activities such as interprofessional case discussions could be conducted with students at other locations using secure communication platforms. During the COVID-19 pandemic, healthcare educators developed greater facility with online platforms [59] and positive outcomes in terms of IPE are emerging [60].

Clarifying why and how to implement practice-base IPE enhances its perceived value. Maintaining patient care and safety is a key activity objective for clinical educators and students. Therefore, activity that is framed as supporting this objective is likely to be perceived as having greater value. To this end practice-based IPE may benefit from a greater focus on its role in improving patient safety and quality of care [61], in addition to the educational benefits as this is an objective with high value for healthcare staff and students. A common misconception reported was that practice-based IPE requires novel, time-intensive activities. However, our findings indicate that brief activities such as interprofessional tutorials or a joint assessment session with appropriate resources to support interprofessional learning can have a meaningful impact. This aligns with recommendations that practice-based IPE is more sustainable if it can be achieved efficiently without requiring substantial resource allocation [62]. Applying the cultural lens, initially adopting small-scale projects may address hesitancy round moving away from traditional models (uncertainty avoidance) [35].

Participants in this study noted that ‘*passive observation’* of other students would have been less impactful than activity guided by the interprofessional observation template, which focused their attention. This echoes previous graduate feedback that effective interprofessional learning during placement needed structure and focus [8]. Consequently, two key features are extrapolated for educators seeking to develop sustainable practice-base IPE. First, liaise with other educators to consider what reasonable adaptions could be made to support authentic IPE opportunities during student’s placement day. Culturally, this can allay concerns regarding relinquishing established placement practices (uncertainty avoidance), whilst ensuring activity is meaningful for both students and patients. Second, utilise appropriate tools to guide and capture learning from these activities. While templates from a range of countries are available [63], developing or adapting tools in conjunction with clinical educator colleagues and the placing university can ensure alignment with locally available opportunities and assessment tools. This can evidence the learning gained from practice-based IPE in real time. Pedagogically, guided activity and reflection creates a robust learning experience and may be particularly beneficial in cultures where there is a preference for achieving outcomes relatively quickly (short-term orientation) [35].

Currently the link between practice-based IPE and learning outcomes is tenuous, as competencies relating to practice-based IPE are broadly framed. Placement providers do require flexibility to deliver practice education in line with specific programme requirements and local capacity. However, ambiguous phrasing of expectations can lead to a policy-practice chasm between what is perceived to be occurring based on formal documents and what is actually happening in practice [64]. The danger with this situation is that complacency may set in, with the rhetoric of practice-based IPE in the absence of meaningful integration into curriculums. Based on current research it is recommended that dedicated practice-based IPE competencies and guidelines are developed. While this would require collaborative working and national level agreement by regulators, higher educational institutions, and placement providers, it would represent significant progress in embedding practice-based IPE in healthcare curriculums. Embedding detailed expectations in documents with regulatory approval may help educators justify this activity [65].

Most IPE educator research to date has focused on university faculty [66, 67]. However, clinical educators represent a more diverse group [68]. They continue to hold core clinical roles and are not centrally organised as an educational team. Developing clinical teaching teams introduces the idea that educators across professions could contribute to student education [69], promoting educator networks at clinical sites. This may help address the issue of reliance on individuals or small groups of champions for practice-based IPE, creating a community of educators who can share the division of labour. Involvement of organisational leadership in developing these networks could provide essential support for increasing visibility and status of practice-based IPE. In practical terms offering interprofessional facilitation training would both support educators to work with students from other professions [3], while also evidencing organisational investment and value in practice-based IPE. Furthermore, placement sites often host students from different institutions, which may facilitate opportunities for inter-institutional practice-based IPE [70]. While this would require agreement at a national level between host universities and placement providers, it may broaden opportunities for practice-based IPE and diversity of professional involvement [70]. Initially, convening an inter-institutional, interprofessional steering group is recommended, with student, university, regulatory and placement-provider representatives. This group could develop governance guidelines and support an initial action plan for trialling this type of practice-based IPE [71].

Beyond the level of individuals and local placement sites, national cultural preferences can shed light on how practice-based IPE may be perceived and valued [34]. This in turn may help tailor the approach to integrating practice-based IPE on a country-by-country basis. In this research adapting existing practice education activities was preferred over introducing wholly new models for practice-based IPE. Hofstede reported that Irish culture tends to prefer normative and traditional ways of operating. Similarly, Bonello and Morris [72] considered the introduction of IPE to Maltese healthcare curriculums through the lens of Hofstede’s cultural dimensions. They found that participant data reflected the national preference for uncertainty avoidance, which was useful to account for when implementing IPE. While data from individuals or groups cannot be assumed to represent overall culture [73] and cultural tendencies should not be perceived as predictive [74], they can draw attention to less visible factors impacting the integration of models such as practice-based IPE across countries [75].

Limitations in the breadth of data from which recommendations were generated warrants consideration. Educators were from one clinical site and student experiences of practice-based IPE was primarily at this site. There did not appear to be factors significantly differentiating this site from typical healthcare placement sites. However, considering the cultural research orientation it cannot be discounted that site specific or local factors were influential. The context of the study allowed for immersion in staff and students experiences and detailed analytical consideration of embedding practice-based IPE, which is appropriate for a case study. Two other sites were to be included but this was not feasible due to COVID-19 pandemic. Subsequent studies could build on this research to include other acute and community sites, to develop a comprehensive profile of practice-based IPE and understand core features required for establishing culturally relevant practice-base IPE.

At the time of writing the ongoing COVID-19 global crisis has highlighted the need for a flexible and collaborative workforce [76], however, it does not automatically resolve pre-existing challenges and may perpetuate some issues [77]. Regarding practice-based IPE, there may a risk of reverting to uniprofessional silos to achieve perceived core uniprofessional competencies. Future planning for practice-based IPE may require even closer collaboration with placement providers.

## Conclusions

Practice-based IPE offers authentic opportunities to develop collaborative working skills [5]. This paper draws on student and clinical educator experiences to offer recommendations for enhancing the value and sustainability of practice-based IPE. Clarifying the concept of practice-based IPE, clearly mapping activities on measurable competencies, and developing diverse educator networks would support embedding of this model and add to its value would support embedding of this model and add to its value. Prevailing local and national cultures should be considered when developing implementation strategies [72]. Crucially, impactful practice-based IPE does not necessitate overhauling practice education. Rather, thoughtful and explicit adaptations to existing practices can lead to meaningful outcomes for students and sustainable models of practice-based IPE.

## Supplementary Information

**Additional file 1.**

**Additional file 2.**

**Additional file 3.**

**Additional file 4.**

**Additional file 5.**

## Data Availability

The corresponding author, Noreen O’Leary, can be contacted with queries relating to data. The datasets (observational notes and interview transcripts) are not publicly available to maintain participant privacy.

## Abbreviations

IPE: Interprofessional education
IPC: Interprofessional collaboration
