# Supplementary figures and images for: ‘It benefits patient care’: the value of practice-based IPE in healthcare curriculums
Source: BMC Med Educ. 2020 Nov 12;20:424. doi: 10.1186/s12909-020-02356-2 (PMC7658912; doi:10.1186/s12909-020-02356-2)

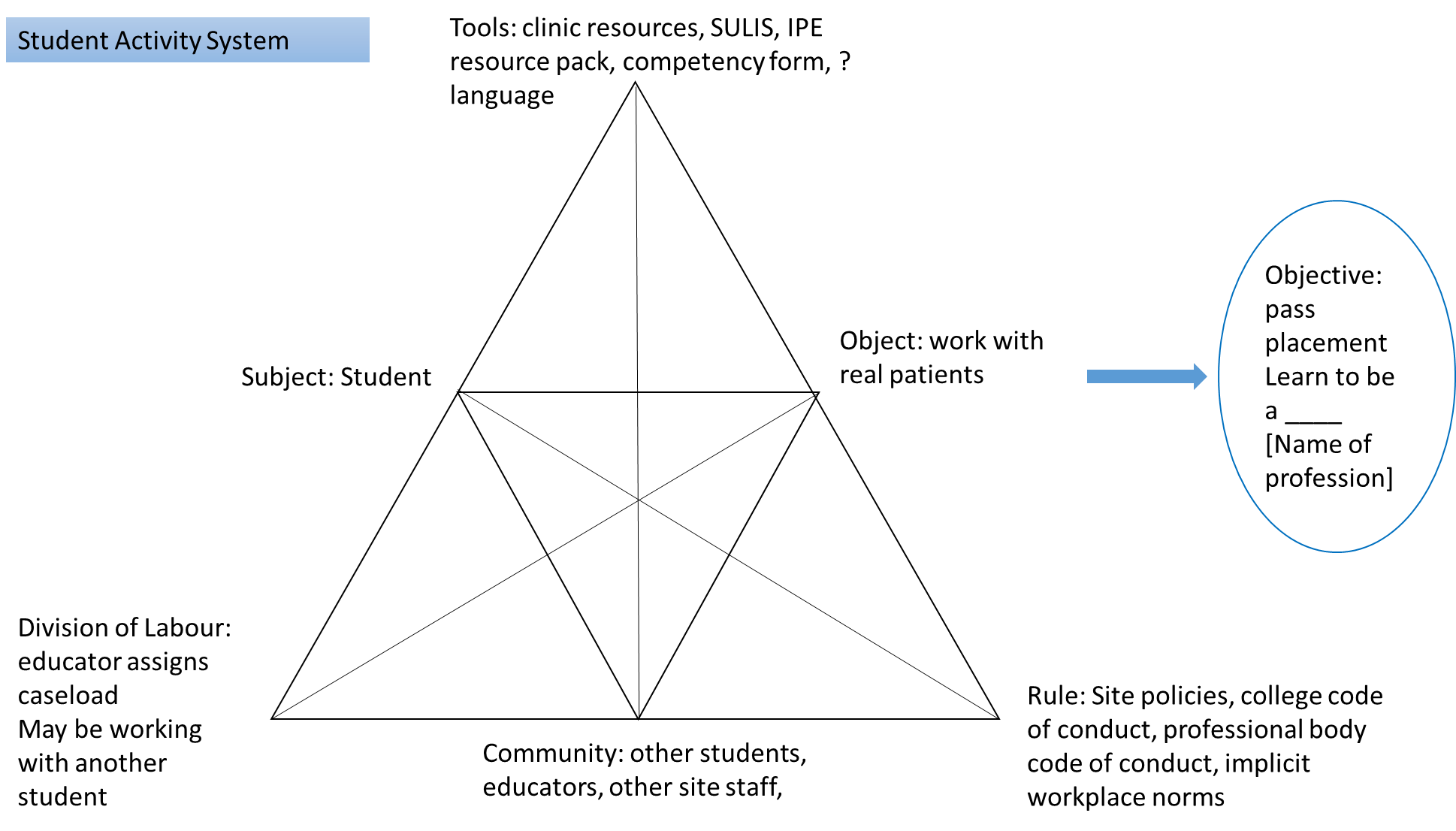


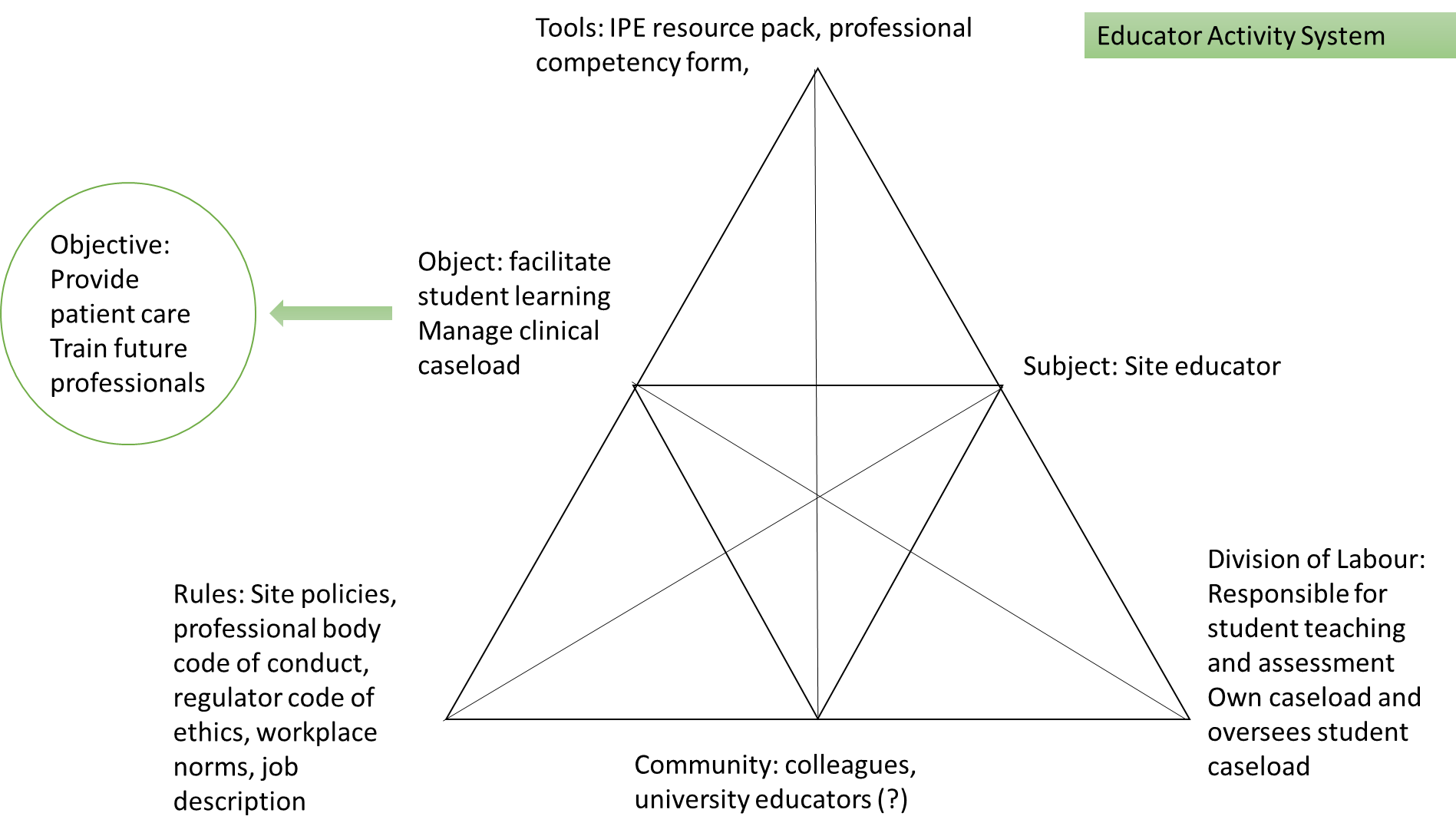


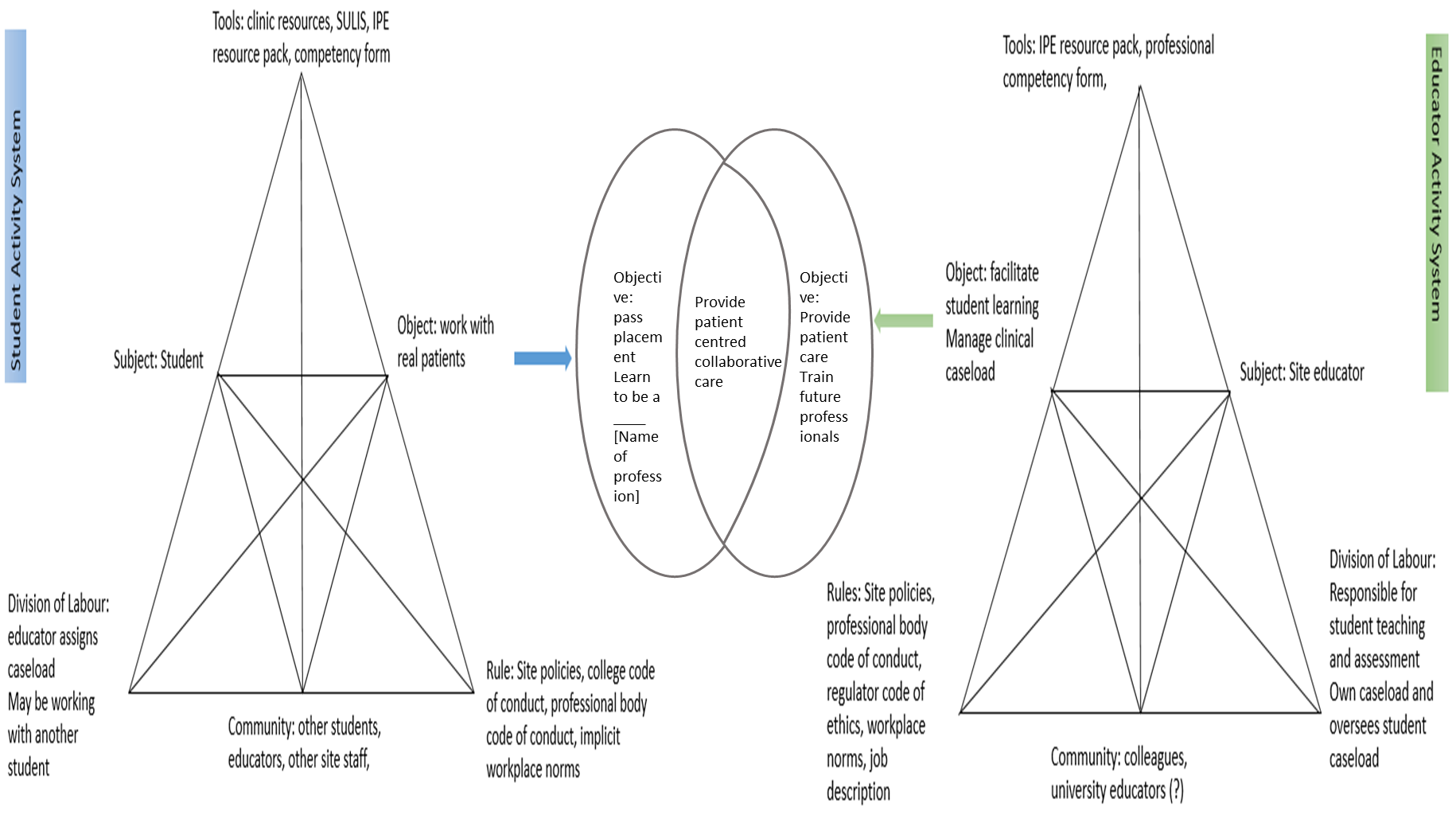

Supplement: Supplementary file 1 — Additional file 1. [file 12909_2020_2356_MOESM1_ESM.docx]
