# Supplementary material for: ‘It benefits patient care’: the value of practice-based IPE in healthcare curriculums
Source: BMC Med Educ. 2020 Nov 12;20:424. doi: 10.1186/s12909-020-02356-2 (PMC7658912; doi:10.1186/s12909-020-02356-2)
Supplement: Supplementary file 2 — Additional file 2. [file 12909_2020_2356_MOESM2_ESM.docx]

Drawing on large scale international surveys conducted in 1967-1973, 1991 and 2010^[[1]](#endnote-1)^, Hofstede identified six dimensions underpinning national cultures and which can be compared cross culturally^[[2]](#endnote-2)^. For each dimension a country is given a score of 0-100, reflecting the national tendency relating to that dimension^[[3]](#endnote-3)^. A score below 50 reflects a relatively low score and a score of over 50 reflects a high score on that dimension^[[4]](#endnote-4)^.

| **Dimension** | **Ireland Score** | **Interpretation** |
| --- | --- | --- |
| Power Distance considers democracy and power distribution within a society. | 28 | Irish culture values a democratic society. |
| Uncertainty Avoidance considers degree of openness to innovation and desire for structure within a society. | 35 | Irish culture prefers to maintain tradition and certainty |
| Individualism versus Collectivism considers the level of integration and dependence between groups in a society. | 70 | Irish culture tends towards individualism |
| Masculinity versus Femininity considers whether a society tends to prioritise achievement or nurturing. | 68 | Irish culture is achievement and outcome orientated. |
| Long Term versus Short Term Orientation considers attitude to tradition and speed of results. | 24 | Irish culture is normative (values tradition) and immediate results |
| Indulgence versus Restraint considers attitude towards enjoying life and desire gratification in a society. | 65 | Irish culture values free time and enjoyment of life |

1. Hofstede G, Hofstede GJ, Minkov M. Cultures and organizations: software ofthe mind: intercultural cooperation and its importance for survival. . New York: McGraw-Hill; 2010. [↑](#endnote-ref-1)
2. Borg MA. National cultural dimensions as drivers of inappropriate ambulatory care consumption of antibiotics in Europe and their relevance to awareness campaigns. *The Journal of antimicrobial chemotherapy*. 2012;67(3):763-7. [↑](#endnote-ref-2)
3. Beugelsdijk S, Welzel C. Dimensions and Dynamics of National Culture: Synthesizing Hofstede With Inglehart. *Journal of cross-cultural psychology*. 2018;49(10):1469-505. [↑](#endnote-ref-3)
4. Bonello M, Morris J, Azzopardi Muscat N. The role of national culture in shaping health workforce collaboration: Lessons learned from a case study on attitudes to interprofessional education in Malta. *Health Policy*. 2018;122(10):1063-9. [↑](#endnote-ref-4)
