# Supplementary material for: ‘It benefits patient care’: the value of practice-based IPE in healthcare curriculums
Source: BMC Med Educ. 2020 Nov 12;20:424. doi: 10.1186/s12909-020-02356-2 (PMC7658912; doi:10.1186/s12909-020-02356-2)
Supplement: Supplementary file 4 — Additional file 4. [file 12909_2020_2356_MOESM4_ESM.docx]

**Participant Observations**

| **Describe the observation environment in detail** | | |
| --- | --- | --- |
| **Describe the participant(s) in detail** | | |
| **Describe the observation process** (e.g. flow, depth of participant responses, rapport between observer and participant, change over the course of the observation). | | |
| **Think back over the observation. Were there any keywords or phrases used by the participant that struck you in some way? If so, list them here.** | | |
| **Summarize the key points from this observation in 2-3 paragraphs.** | | |
| **How does this observation address / go towards answering the research question?** | | |
| **Describe how this observation connects to the research aims.** | | |
| **Reflect on your own experience of the observation itself. How did you respond throughout the session? Did you hear pretty much what you expected to hear? If so, explain. Did anything about the participant’s experience surprise you or make your feel uncomfortable? If so, explain.** | | |
| **Activity theory** | | |
| **Subject** | **Object** | **Community** |
|  |  |  |
| **Division of Labour** | **Rules** | **Tools** |
|  |  |  |

**Interview Schedule**

Student interview schedule

| **Question** |
| --- |
| 1. As a student in a healthcare programme, what does interprofessional placement mean to you? |
| 1. Did you have any preparation for working interprofessional before going on placement? |
| 1. During your placements have you had any experience of working interprofessionally? If so, please describe those experiences.   **OR:**  If you haven’t had any interprofessional experiences how do you feel about that? |
| 1. What was the most useful learning from your interprofessional experiences?   **OR**  What would you hope to learn / gain from interprofessional placement experiences |
| 1. What challenges arose when working in this way?   **OR**  Can you imagine / foresee any challenges to interprofessional working on placement? |
| 1. How do you feel about interprofessional placements being part of the curriculum for your course? |
| 1. In terms of setting up IPP, what would make for a ‘good IPP’ for you as a student or for future students? [key student activities, length of time, range of professions] |
| 1. Do you foresee interprofessional placement experiences influencing your future practice [in what ways do you see this changing your practice?] |

Thank you for your time and participation in this interview

Educator interview schedule

**Introduction:** I know that here in SAH lots of people have been involved in planning, supporting and reflecting on IPP over the last few years. I am interested in hearing about the range of experiences from people who have had different types of involvement.

| Can you tell me about your own involvement/journey with interprofessional placements (IPP)? [have you been involved in an interprofessional placement, how do you see IPP unfolding within SAH if no involvement to date] |
| --- |
| 1. What is your biggest driver for IPP? |
| How would you go about setting up an IPP? (query overall process, people to liaise with, documentation required) |
| 1. What would be your biggest concerns about IPP?   [Of concerns / challenges, would any of them stop you going ahead with an IPP] |
| What strategies do you use to inform your decisions about IPP? (query tools/ regulations/ guidelines) |
| Where does IPP fit within the overall scheme/ demands/ priorities of your work as an ________ [role} in SAH? [] |
| 1. When you ran an IPP what did it look like? [in terms of what activities students did / how they interacted with patients / length of IPP]    1. OR   What would an IPP look like if you were running one? |
| 1. Reflecting on your IPP experiences what was the main outcome (positive/negative) for:    1. You as an educator    2. Students [ are assessment tools capturing student learning    3. Patients |
