# Supplementary material for: ‘It benefits patient care’: the value of practice-based IPE in healthcare curriculums
Source: BMC Med Educ. 2020 Nov 12;20:424. doi: 10.1186/s12909-020-02356-2 (PMC7658912; doi:10.1186/s12909-020-02356-2)
Supplement: Supplementary file 5 — Additional file 5. [file 12909_2020_2356_MOESM5_ESM.docx]

**Data Analysis**

**Thematic Analysis**


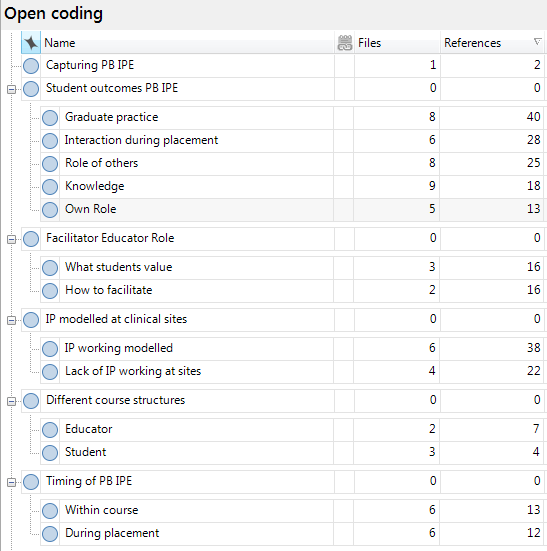


*Extract of open coding*


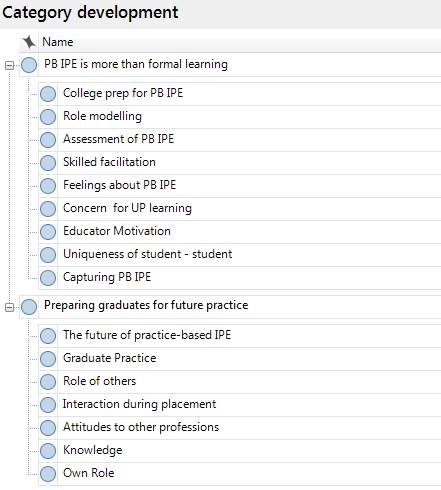


*Category Development*

**Framework Analysis**


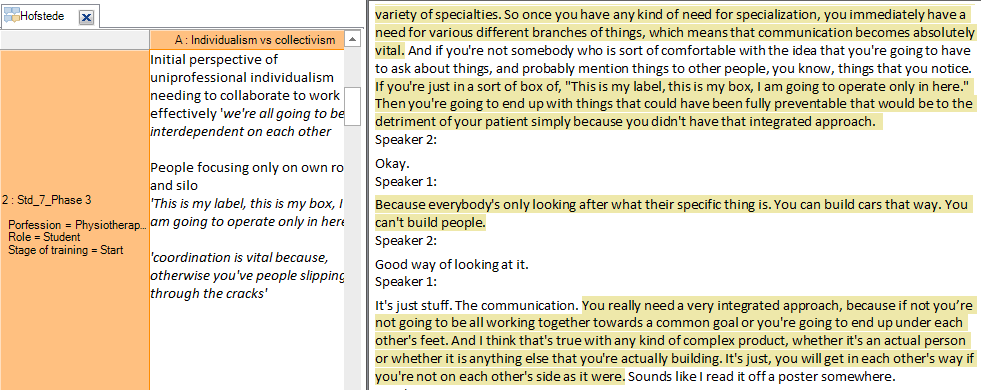


*Data summary extract*
